# Insights into the Bacterial and Nitric Oxide-Induced Salt Tolerance in Sugarcane and Their Growth-Promoting Abilities

**DOI:** 10.3390/microorganisms9112203

**Published:** 2021-10-22

**Authors:** Anjney Sharma, Rajesh Kumar Singh, Pratiksha Singh, Anukool Vaishnav, Dao-Jun Guo, Krishan K. Verma, Dong-Ping Li, Xiu-Peng Song, Mukesh Kumar Malviya, Naeem Khan, Prakash Lakshmanan, Yang-Rui Li

**Affiliations:** 1 Key Laboratory of Sugarcane Biotechnology and Genetic Improvement (Guangxi), Ministry of Agriculture, Sugarcane Research Center, Chinese Academy of Agricultural Sciences, Guangxi Key Laboratory of Sugarcane Genetic Improvement, Sugarcane Research Institute, Guangxi Academy of Agricultural Sciences, Nanning 530007, China; anjneysharma@gmail.com (A.S.); rajeshsingh999@gmail.com (R.K.S.); singh.pratiksha23@gmail.com (P.S.); gdj0506@163.com (D.-J.G.); drvermakishan@gmail.com (K.K.V.); malviyamm1983@gmail.com (M.K.M.); plakshmanan2018@outlook.com (P.L.); 2 Guangxi Key Laboratory of Crop Genetic Improvement and Biotechnology, Nanning 530007, China; xiupengsong@gxaas.net; 3 Department of Biotechnology, GLA University, Mathura 281406, U.P., India; anukoolv7@gmail.com; 4 College of Agriculture, State Key Laboratory of Conservation and Utilization of Subtropical, Agro-Bioresources, Guangxi University, Nanning 530005, China; 5 Microbiology Institute, Guangxi Academy of Agricultural Sciences, Nanning 530007, China; lidongping0201@126.com; 6 Department of Agronomy, Institute of Food and Agricultural Sciences, University of Florida, Gainesville, FL 32611, USA; naeemkhan@ufl.edu; 7 Interdisciplinary Research Center for Agriculture Green Development in Yangtze River Basin, College of Resources and Environment, Southwest University, Chongqing 400715, China; 8 Queensland Alliance for Agriculture and Food Innovation, The University of Queensland, St. Lucia, QLD 4072, Australia

**Keywords:** soil salinity, PGPR, sodium nitroprusside, synergistic effects, reactive oxygen species, antioxidant enzyme, sustainable agricultural production

## Abstract

Soil salinity causes severe environmental stress that affects agriculture production and food security throughout the world. Salt-tolerant plant-growth-promoting rhizobacteria (PGPR) and nitric oxide (NO), a distinctive signaling molecule, can synergistically assist in the alleviation of abiotic stresses and plant growth promotion, but the mechanism by which this happens is still not well known. In the present study, in a potential salt-tolerant rhizobacteria strain, ASN-1, growth up to 15% NaCl concentration was achieved with sugarcane rhizosphere soil. Based on 16S-rRNA gene sequencing analysis, the strain ASN-1 was identified as a *Bacillus xiamenensis*. Strain ASN-1 exhibits multiple plant-growth-promoting attributes, such as the production of indole-3-acetic acid, 1-aminocyclopropane-1-carboxylate deaminase, siderophores, HCN, ammonia, and exopolysaccharides as well as solubilized phosphate solubilization. Biofilm formation showed that NO enhanced the biofilm and root colonization capacity of the PGPR strain ASN-1 with host plants, evidenced by scanning electron microscopy. The greenhouse study showed that, among the different treatments, the combined application of PGPR and sodium nitroprusside (SNP) as an NO donor significantly (*p* ≤ 0.05) enhanced sugarcane plant growth by maintaining the relative water content, electrolyte leakage, gas exchange parameters, osmolytes, and Na^+^/K^+^ ratio. Furthermore, PGPR and SNP fertilization reduced the salinity-induced oxidative stress in plants by modulating the antioxidant enzyme activities and stress-related gene expression. Thus, it is believed that the acquisition of advanced information about the synergistic effect of salt-tolerant PGPR and NO fertilization will reduce the use of harmful chemicals and aid in eco-friendly sustainable agricultural production under salt stress conditions.

## 1. Introduction

Abiotic stresses are the most common environmental constraints to agricultural production and food security around the world. It is estimated that environmental abiotic stresses such as salt, drought, high/low temperature, heavy metals, and chemical pesticides affect 90% of all arable land [1]. Among these abiotic stresses, soil salinity, caused mainly by groundwater irrigation, is one of the most serious abiotic stresses for plant growth and agricultural production [2]. Due to the increasing soil salinity, decreasing agricultural lands might be a severe problem for food supply for the increasing world population in the near future [3,4]. It has been estimated that at least 50% of the world’s arable land will be salt-affected by the year 2050, and this number is expected to rise due to global climate change [5]. As a result of soil salinity and sodicity, about 70% of plant growth and production loss have been documented in various crops [6]. The major cations, such as Na^+^ (sodium), Ca^2+^ (calcium), and Mg^2+^ (magnesium), and anions, such as Cl^−^ (chloride), SO_4_^2−^ (sulfate), CO_3_^2−^ (carbonate), and bicarbonate (HCO_3_^−^), are present in the total soluble salts of saline soils [7]. Among all constituents of soluble salts, the cation Na^+^ and anion Cl^−^ are metabolically toxic to crops [8]. A higher salt concentration (>50 mmol L^−1^) imposes deleterious adverse effects on agronomic crops, inhibiting a variety of physiological, biochemical, and metabolic processes [9]. Plants exposed to salt stress can experience membrane lipid peroxidation, cellular redox homeostasis disruption, pigment bleaching, protein degradation, enzyme inactivation, and macromolecule destruction [10]. In the later stage of salt stress, the accumulation of Na^+^ and Cl^−^ ions altered the Na^+^/K^+^ and Na^+^/Ca^2+^, which induced ionic stress and ultimately promoted the generation of reactive oxygen species (ROS) and leads oxidative stress in cells [5,11,12]. In the different locations of plant cells, the ROS molecules [superoxide anion (O_2_^−^), singlet oxygen (^1^O_2_), and hydrogen peroxide (H_2_O_2_)], lowered the photosynthetic rate, which affects the electron flow and consequently leads to oxidative burst, plant injury and cell death [13]. Additionally, intracellular ion imbalance obstructs soil nutrient uptake, leading to nutrition deficiencies [14].

To mitigate the salt stress, many chemical fertilizers and pesticides, as well as traditional breeding and genetic engineering procedures, have shown little success in generating salinity-resistant plants [9,15,16,17]. Furthermore, these strategies are not viable at the field level due to a variety of factors, and adequate, sustainable results have not been obtained through biological means [18]. As a result of these problems, there is an urgent requirement to develop novel techniques to alleviate salt stress and improve crop yield in such difficult soils in order to feed the world’s growing population with nutritious food. Exploiting salt-tolerant PGPR for a sustainable environment and safe agriculture has recently emerged globally as a viable technique for addressing the aforementioned scenario [5,10]. Salt-tolerant PGPR survive or adopt the stress condition by the de novo synthesis of osmolytes, maintain homeostasis, and regulate the ionic transporters to reduce the harmful toxic effects of Na^+^ and Cl^−^ ions [19]. To mitigate the brutal abiotic and biotic environmental stresses and subsequent harmful yield consequences, the salt-tolerant PGPR employ their several synergistic direct and indirect mechanisms to protect the host plant [20,21]. Through the direct mechanism, PGPR facilitate essential nutrients such as nitrogen, phosphate, zinc, potassium, and iron and the production of ACC deaminase and auxin-like phytohormones [2,22,23]. Through an indirect mechanism, PGPR prevent the harmful effects of pathogens and promote plant growth by producing siderophores, HCN, and antimicrobial metabolites [23,24]. Recently, microbial inoculation has been used to understand plant responses against salt stress, and the results of these studies suggested that osmolyte accumulation (glycine betaine, proline, sugars, etc.) and the enzymatic antioxidative defense system [SOD (superoxide dismutase, EC 1.15.1.1), CAT (catalase, EC 1.11.1.6), POD (peroxidase, EC 1.11.1.7), APX (ascorbate peroxidase, EC 1.11.1.11), PPO (polyphenol oxidase, EC 1.10.3.1), and LOXs (lipoxygenases, EC 1.13.11.12)] detoxify and neutralize the salinity-induced excessive amount of ROS. Thus, this helps in maintaining the defense in response to oxidative damage and increases the plant’s resistance under induced osmotic stress [2,9,10]. Several bacterial species represented by the genera *Agrobacterium*, *Bacillus*, *Enterobacter*, *Klebsiella*, *Ochromobacter Pseudomonas*, *Rhizobium*, *Serratia*, *Sphingobacterium*, and *Stenotrophomonas*, and *Streptomyces* species have been widely recognized as effective bio-inoculants in salinity-affected agriculture [13,24,25].

Furthermore, seed treatment with various inorganic and organic compounds prior to planting is thought to be a significant strategy to improve plant tolerance to adverse environments [26]. Of the different seed priming agents, the exogenous application of nitric oxide (NO) is an economical method in protecting plants from salt stress [27,28]. Nitric oxide (NO), which is small in size, has no charge, and it is a diatomic free radical gaseous molecule with a short half-life; it is highly diffusible across biological membranes, can effectively mitigate osmotic stress by enhancing antioxidant enzyme activities, maintain the Na^+^/K^+^ ratio, provoke a variety of physiological and biochemical responses, and enhance plants grown under salinity stress and other abiotic stresses [28,29,30]. Nevertheless, NO pre-treatment boosted the NaCl-induced post-translational modifications (PTMs), such as protein S-nitrosylation and tyrosine nitration, which have an influence on the activity of different enzymes that perform a defensive role under abiotic stress conditions [31]. There is increasing evidence showing that NO has several extra properties that make it a signal molecule for regulating various processes such as plant growth and development, plant–water relations, respiratory metabolism, floral regulation, seed germination, the biosynthesis of photosynthetic pigments, photosynthetic efficiency, stomatal conductance, fruit ripening, senescence cell death, ion leakage, biomass production, and the formation of adventitious roots under stressed and unstressed conditions [32,33,34,35].

Sugarcane (*Saccharum officinarum* L.) is a key, economically important, cash crop grown throughout the tropical and subtropical regions of the world [36]. However, since it is sessile in nature, sugarcane is highly vulnerable to salinity stress (EC > 2 dSm^−1^), and excessive salt concentration causes toxicity symptoms that directly or indirectly affect its physiological and metabolic process as well as its nutritional value and also reduce sugarcane growth (15–86%) [37,38,39,40]. An earlier attempt to confer salt tolerance in sugarcane through PGPR inoculation has been made [41]. However, the mechanism by which PGPR and NO facilitate salt stress tolerance in sugarcane plants has not been investigated. Thus, salt-tolerant, PGPR from the sugarcane rhizosphere were explored in this study, while their effects, alongside SNP as an NO donor on the mitigation of salt stress on sugarcane, were evaluated by modulating the physiological, biochemical, and antioxidant enzyme activities and stress-responsive gene expression. This research extends our knowledge of salt-tolerant, PGPR-mediated, systemic abiotic stress tolerance in plants and encourages us to use microorganism inoculants for salt land reclamation.

## 2. Materials and Methods

### 2.1. Collection of Soil Samples and Isolation of Rhizobacteria

**Figure 1:**
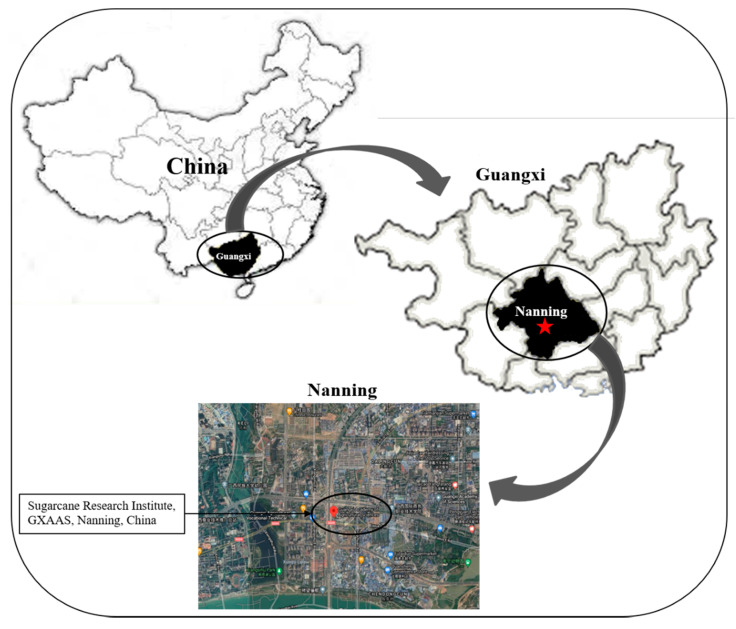
The location of the experimental site.

Rhizosphere soil samples from the widely grown sugarcane variety GT44 were collected in the month of June 2020 from the agriculture field of the Sugarcane Research Institute, Guangxi Academy of Agricultural Sciences, Nanning, Guangxi, China (Latitude 22°50′ N; Longitude 108°14′ E) (Figure 1). The study area has a humid subtropical climate with an annual mean temperature of 21.83 °C and a rainfall of 1290 mm. The collected rhizosphere soil was sandy, silty, and clayey in texture with a 7.2 pH and a 3.2 dSm^−1^ electric conductivity (EC). For the isolation of salt-tolerant rhizobacteria, a dry and sieved composite rhizosphere soil sample was enriched in NaCl (10%)-supplemented nutrient broth (NB) for 5 days at 28 °C. Afterward, a serially diluted (10^−3^, 10^−4^, and 10^−5^) enriched soil sample was spread on nutrient agar (NA) plates supplemented with 10% NaCl and incubated for 48 h at 28 °C. After incubation, a fast-growing bacterial colony (ASN-1) showing dominant growth was picked, purified by sub-culturing on a fresh NA plate, and preserved in 50% glycerol at −80 °C for further use.

### 2.2. Intrinsic Salt (NaCl) Tolerance Assay

To evaluate the NaCl tolerance efficiency, 1% of fresh, exponentially grown bacterial culture was inoculated in NB supplemented with different concentrations (5, 10, 15, and 20%) of NaCl and incubated in an incubator at 28 °C for 72 h at 150 rpm. The growth of the isolate at various levels of NaCl stress was analyzed by measuring the optical density (OD) in a spectrophotometer (Shimadzu, Kyoto, Japan) at 600 nm at every 12 h time interval.

### 2.3. Morphological and Biochemical Characterization

Morphological and biochemical features of the selected bacterial strain ASN-1 were studied by following Bergey’s Manual of Determinative Bacteriology [42]. Culture grown on a nutrient agar medium at 28 ± 2 °C was examined for its cellular morphology and gram reaction. Subsequently, a strain was metabolically characterized based on its carbon source utilization pattern using a BIOLOG GNIII MicroPlate™ (Biolog, Inc., Hayward, CA, USA) following the manufacturer’s protocol.

### 2.4. Screening of Plant-Growth-Promoting (PGP) Attributes and Exopolysaccharide Production

The selected salt-tolerant bacterial strain, ASN-1, was screened for different PGP attributes, such as the production of IAA, ACC deaminase, siderophores, HCN, ammonia, and exopolysaccharide (EPS) and phosphate solubilization, under normal (no NaCl stress), 5%, and 10% NaCl stress conditions.

#### 2.4.1. Indole-3-Acetic Acid (IAA) Production

IAA production was screened by adopting the method of Gupta and Pandey [43]. Briefly, a freshly grown 1% bacterial culture was inoculated into sterile nutrient broth (10 mL) and incubated at 28 ± 2 °C for 48 h. After incubation, the culture was centrifuged, and cell-free supernatant was collected. Two milliliters of culture supernatant, 2–3 drops of glacial acetic acid, and 4 mL of Salkowski reagent (50 mL of 35% perchloric acid and 1 mL of a 0.5 M FeCl_3_ solution) were added and incubated at room temperature for 60 min under dark conditions. After incubation, the appearance of pink in the solution indicated IAA production. The optical density of the pink-colored reaction mixture was taken at 530 nm using a UV spectrophotometer. The quantity of IAA production was determined through a standard calibration curve of pure IAA (10–100 mg/mL).

#### 2.4.2. ACC Deaminase Activity

The ACC deaminase activity was tested by adopting the protocol of Misra et al. [44]. Pure bacterial culture was spot-inoculated on a DF salt minimal medium plate [45], augmented with 3 mM ACC as the only source of nitrogen, and incubated at 28 ± 2 °C for one week. The plate containing DF salt minimal medium without any nitrogen source served as the negative control. After the incubation time, the bacterial growth on the DF salt minimal agar plate supplemented with 3 mM ACC showed ACC deaminase activity.

#### 2.4.3. Phosphate (P) Solubilization

The phosphate solubilization capacity of the bacterial strain was assayed by spot inoculation of fresh bacterial culture on Pikovaskaya’s agar medium plate [46], and plates were incubated at 28 ± 2 °C for one week. After the positive incubation result of phosphate, solubilization was observed as a halo zone around the bacterial growth [47].

#### 2.4.4. Siderophore Production

Siderophore production was estimated by the spot inoculation of actively grown bacterial culture on a Chrome azurol S (CAS) agar medium plate following a method used by Singh and Jha [48]. The bacterial inoculated plates were incubated for 72 h at 28 ± 2 °C, and after the incubation time, the formation of a yellow to orange halo zone around the bacterial growth indicated siderophore production.

#### 2.4.5. Hydrogen Cyanide (HCN) Production

The production of HCN was performed by following the method of Singh et al. [49]. Fresh bacterial culture was streak inoculated on a King’s B medium plate supplemented with 4.4 g glycine L^−1^. Afterward, sterilized filter paper pre-soaked in a solution of picric acid (0.5%) and Na_2_CO_3_ (2%) was fixed to the upper part of the inoculated medium plates and incubated for 72 h (28 ± 2 °C). After incubation, a change in the color of the filter paper from yellow to brown or orange was considered evidence of HCN production.

#### 2.4.6. Ammonia Production

Ammonia production was investigated by following the method of Dey et al. [50]. Actively grown bacterial culture was inoculated in peptone water (10 mL) and incubated at 28 ± 2 °C for 48 h. Afterward, 0.5 mL of Nessler’s reagent was mixed in the tube, and a change in appearance in the reaction mixture from deep yellow to a brownish color indicated ammonia production.

#### 2.4.7. Exopolysaccharide (EPS) Production Assay

EPS production of the selected bacterial strain was tested by adopting the method of Kumari et al. [51]. Briefly, bacterial culture was grown in 100 mL of NB media supplemented with or without 5% and 10% NaCl and incubated at 37 °C for 96 days at 150 rpm. After the incubation, the EPS fraction was extracted from the centrifuged bacterial supernatant by adding three volumes of pre-chilled absolute ethanol, and the formation of a precipitate indicated EPS production.

### 2.5. Analysis of Biofilm Formation

The biofilm formation of the selected bacterial culture was measured by following the method of Qurashi and Sabri [52]. A bacterial cell suspension of 10^8^ CFU ml^−1^ was prepared in NB medium. Afterward, 0, 5, and 10% NaCl as well as 100 µM SNP were added to the cell suspension. Subsequently, 200 µL of this treated bacterial suspension was transferred to a 96-well microliter plate in five replications. The plate was sealed and then adequately incubated at 37 °C for 48 h. The un-inoculated NB medium with 0, 5, and 10% NaCl and 100 µM SNP served as a control. After the incubation time, the growth medium from each well of the microliter plate was gently discarded. The well was washed 3–4 times with sterile D/W (distilled water) to eliminate liberated cells of the bacteria. After washing, a 0.01% crystal violet dye was added to each dried well, and stained wells were again rinsed with sterile D/W 2–3 times and air-dried. The remaining dye was solubilized by adding 100 µL of acetic acid (30%). Quantification of the biofilm formation was carried out spectrophotometrically by taking the absorbance at 570 nm.

### 2.6. Molecular Identification Based on 16S rRNA Gene Sequencing Analysis

Genomic DNA from the bacterial isolate-1 was extracted by following the method of Sharma et al. [53]. For the amplification of the 16S rRNA gene, a universal 16S rRNA gene-specific primer set, namely, PA (5′-AGA GTT TGA TCC TGG CTC AG-3′) and PH (5′-AAG GAG GTG ATC CAG CCG CA-3′), was used [23]. The amplification was carried out in a total 100 µL reaction mixture comprising a 10X reaction buffer, 2.5 mM dNTPs, 10 pM of each primer, 3 U Taq polymerase, and template DNA (50 ng). The amplification was performed in a thermal cycler (Bio-Rad, Hercules, CA, USA) under the following PCR conditions: initial denaturation at 94 °C for 4 min, followed by 35 cycles of denaturation at 94 °C for 45 s, annealing at 52 °C for 45 s, and elongation at 72 °C for 90 s, with a final extension period of 7 min at 72 °C. The amplified 16S rRNA gene PCR product was purified using a BioFlux PCR purification kit (Bioer Technology Co. Ltd., Hangzhou, China). The purified PCR product was sequenced using the Applied Biosystems (ABI- Prism, Waltham, MA, USA) automated DNA sequencer (Sangon Biotech, Shanghai, China). The obtained 16S rRNA gene sequence of the bacterial strain ASN-1 was aligned using CLUSTAL-W with available 16S rRNA gene sequences of the GenBank database. A neighbor-joining (NJ) phylogenetic tree was created through MEGA-X by evolutionary distance calculations and the Jukes–Cantor coefficient [54,55]. The phylogenetic tree topology was assessed through the bootstrap resampling method with 1000 replicates [56].

### 2.7. Greenhouse Study for Plant Growth Promotion

The greenhouse experiment was performed to evaluate the synergistic effect of the salt-tolerant bacterial strain ASN-1 and SNP (as a NO donor) for growth promotion and induced systematic tolerance of sugarcane under salt stress conditions (Sugarcane Research Institute, Guangxi Academy of Agricultural Sciences, Nanning, China). The bacterial suspension was prepared by growing the bacterial culture in NB medium on a rotary shaker at 28 °C at 150 rpm for 48 h. After the incubation through centrifugation (at 12,000 rpm for 10 min at 28 °C), the bacterial cells were collected, and washed 2–3 times with sterile D/W, and resuspended in 0.1 M phosphate buffer (pH 7.0) to achieve a cell density of ~10^8^ cfu mL^−1^. Fifteen-day-old healthy seedlings of the sugarcane cultivar (GT44) were taken from the nursery of the Sugarcane Research Institute, Nanning, China, and washed 3–4 times with running tap water to eliminate all plants and soil debris. The following eight treatments (T) were used: T-1 (control: no bacterial inoculation, with normal irrigation), T-2 (treated with 100 µM SNP), T-3 (bacterial inoculation), T-4 (100 µM SNP + bacterial inoculation), T-5 (100 mM salt stress), T-6 (100 µM SNP + 100 mM salt stress), T-7 (bacterial inoculation + 100 mM salt stress), and T-8 (100 µM SNP + bacterial inoculation + 100 mM salt stress). For bacterial treatment, the cleaned sugarcane seedlings were absorbed in bacterial suspension for 4 h and then implanted into plastic pots (20 cm × 25 cm) containing 3.5 kg of a sterilized mixture of soil and sand (3:1 *w*/*w*). For SNP treatment, a 100 µM solution of SNP was applied. The seedlings with distilled water and without SNP and bacterial suspension treatment were used as the control. All experimental pots with three induvial replications were arranged in completely randomized manner with a 16/8 h light/dark cycle, a 30 ± 2 °C temperature, and a field water capacity (FWC) of 80% moisture. After 10 days of inoculation and plant establishment, the salt (NaCl) stress (200 mM) was imposed in the plants. After 30 days of constant stress, the sugarcane plants of each treatment were harvested for growth parameters, such as shoot/root length and fresh and dry weight, and for further biochemical and antioxidant defense enzyme analysis. The root and shoot dry weight were evaluated by drying the samples at 65 °C for 96 h in a hot air oven.

### 2.8. Measurement of Photosynthetic Characteristics

Photosynthetic parameters such as net photosynthetic CO_2_ assimilation (Pn; µmol CO_2_ m^−2^ s^−1^), transpiration rate (E; mmol H_2_O m^−2^ s^−1^), and stomatal conductance (gs; mmol H_2_O m^−2^ s^−1^) were observed in the third and fourth fully expanded leaves of each plant using a LICOR-6800 portable photosynthesis system (LI-COR Biosciences, Lincoln, NE, USA). The photosynthetic parameter observations of all treatments were taken during the day between 09:00 and 11:00 a.m., using a photosynthetic photon flux density (PPFD) of 1000 µmol m^−2^ s^−1^, a CO_2_ concentration of 400 ppm, and a leaf temperature of 25 ± 2 °C. All the measurements were taken in triplicate.

### 2.9. Relative Water Content (RWC) Measurement

The RWC of the all-treated plants was measured by following the method of Hasanuzzaman et al. [57]. A 0.1 g (FW) portion of a fully expanded top leaf sample of each treatment was hydrated by soaking it in deionized water for 24 h at room temperature to obtain full turgidity. After the hydration, the leaves were quickly and lightly dried by using filter paper, and the TW (turgid weight) was then obtained. Subsequently, the samples were dried at 80 °C in an oven for 2–3 days. The RWC was determined by using the following formula:

Leaf RWC (%) = (FW − DW)/(TW − DW) × 100

### 2.10. Electrolyte Leakage (EL) Assay

The EL of the leaves of the treated sugarcane plants was measured by adopting the method of El-Tayeb et al. [58]. A 1.0 g portion of the leaf tissue of each treatment was weighed and excised into small leaf discs. The samples were then placed in 10 mL of deionized water for 24 h at 28 °C on an incubator shaker, and with the help of a digital conductivity meter, conductivity was measured as L1. Subsequently, the samples were incubated in a boiling water bath at 120 °C for 20 min. After boiling, the solution was cooled down to room temperature, and electrical conductivity was then measured as L2. The EL (%) was calculated by the following equation:

EL (%) = [1 − (L1/L2)] × 100

### 2.11. Salt Tolerance Index (STI)

The STI of all the treated sugarcane plants was measured by following the method of Al-Garni et al. [59] by using the following formula:

STI (Salt tolerance index) = DWI/DWC

where DWI is the dry weight of the treated plants, and DWC is the dry weight of the non-treated un-inoculated plants.

### 2.12. Analysis of Na^+^/K^+^ Ion Content

The sugarcane leaves were cleaned 2–3 times with Milli Q water and then dried to a consistent weight at 80 °C. To release the free cations, dry powdered leaves samples (0.1 g) were extracted with 5 mL of HNO_3_ (4M) overnight at 37 °C and centrifuged for 10 min at 10,000× *g*. The extracts’ supernatants were diluted, and the concentrations of Na^+^ and K^+^ were measured using an atomic absorption flame spectrophotometer.

### 2.13. Chlorophyll Content (SPAD Meter Values)

The chlorophyll content of the sugarcane leaf of all the treatments was measured through the Soil Plant Analytical Development (SPAD) chlorophyll meter SPAD-502 Plus (KONICA MINOLTA Japan) by following the method described by Jangpromma et al. [60]. The observation was taken from the third fully expanded leaf in replicate on sunny days between 9:00 and 11:00 a.m., and an averaged value was recorded.

### 2.14. Proline Content

The total proline content of the sugarcane leaves was estimated by adopting the method of Kousar et al. [61]. Briefly, 0.1 g leaf samples were ground in 5 mL of 3% sulfosalicylic acid. Glacial acetic acid (2 mL) and 2 mL of ninhydrin acid reagent (20 mL of 6 M phosphoric acid and 1.25 g of ninhydrin in 30 mL glacial acetic acid) was added to 2 mL of the homogenized filtrate, and mixed well. Afterward, the reaction mixture was incubated at a boiling temperature for 60 min. Further, in an ice-cold mixture, 4 mL of toluene was added and mixed vigorously. The chromophore containing toluene was aspirated from the mixture’s aqueous phase, and the optical density was measured at 520 nm. The proline content (mg/g FW) was calculated using L-proline as a standard curve.

### 2.15. Total Soluble Sugar (TSS) Content

The treated plants’ TSS content was determined by following the method of Singh and Jha [62]. A 0.2 g portion of the leaf sample was ground in 10 mL of 80% ethanol (*v*/*v*) and centrifuged for 8 min at 8000 rpm at room temperature (RT). Three milliliters of a fresh anthrone solution (200 mg of anthrone + 100 mL of 72% H_2_SO_4_) was added to 0.1 mL of the extracted supernatant, and the mixture was incubated for 10 min in a boiling water bath. The OD of the cooled reaction mixture was measured with a spectrophotometer at 620 nm, and the TSS concentration (mg/g FW) was calculated using a glucose standard curve.

### 2.16. ROS Scavenging Antioxidant Enzymes Activity Assay

The crude enzyme extract for the estimation of antioxidant enzyme activities was extracted by following the method of Qureshi et al. [63]. In a pre-chilled mortar and pestle, 1.0 g of leaf sample was homogenized with potassium phosphate buffer (100 mM, pH 7.4) comprising 1 mM phenyl-methylsulfonyl fluoride (PMSF), 2% polyvinylpyrrolidone (PVP), and 1 mM EDTA. The mixture was centrifuged for 20 min at 4 °C at 10,000× *g*, and the supernatant acted as an enzyme extract for catalase (CAT), superoxide dismutase (SOD), polyphenol oxidase (PPO), peroxidase (POD), and malondialdehyde (MDA) estimation.

#### 2.16.1. CAT Activity

CAT activity in sugarcane leaves was measured by following a modified method of Qureshi et al. [63]. Fifty microliters of crude enzyme extract, 100 mM potassium phosphate buffer (pH 7), and 20 mM hydrogen peroxide (H_2_O_2_) were added to a 2.5 mL reaction mixture. CAT activity was determined by observing the decrease in absorbance at 240 nm. CAT activity was represented as a breakdown of 1 µmol of H_2_O_2_ per min.

#### 2.16.2. SOD Activity

SOD activity was estimated in terms of the photoreduction of nitroblue tetrazolium chloride (NBT) [63]. The reaction mixture (2.5 mL) consisted of 0.1 M potassium phosphate buffer (pH 7.8), 2 mM NBT, 75 µm riboflavin, 200 mM methionine, 3 mM EDTA, and 50 µL crude enzyme sample. The reaction began at 25 °C under a fluorescent lamp and terminated after 15 min when the lamp was turned off. The identical reaction mixture was held in the dark for the same amount of time for the blank solution. The reduction in NBT was measured by monitoring the alteration in OD at 560 nm. For the calculation of enzyme units (U), readings of the dark blank were used. A one-unit amount of SOD was essential for the photoreduction (50%) of NBT, and SOD activity was expressed in U/mg protein.

#### 2.16.3. PPO Activity

PPO activity was estimated by using the protocol given by Weisany et al. [64]. In a 2.5 mL reaction mixture, 50 µL of enzyme sample, 100 mM potassium phosphate buffer (pH 6), and 1 M catechol was used, and the increase in OD at 420 nm every 30 s for 3 min was recorded. PPO activity was expressed in U/mg protein.

#### 2.16.4. POD Activity

POD activity was spectrophotometrically estimated by adopting the method of Choudhary [65] with a slight modification. The reaction mixture included leaf extract (3 µL) and 3.0 mL of solution consisted of guaiacol (20.1 mM), H_2_O_2_ (12.3 mM), and sodium phosphate buffer (pH 6.0). It was kept in a water bath, and POD activity was then evaluated by taking the absorbance at 436 nm at 30 °C every 30 s for 15 min. The POD enzyme activity was expressed in U/mg protein.

#### 2.16.5. MDA Content

The MDA content of the sugarcane plants of each treatment was measured by using the method of Kumari et al. [51] with minor modifications. Fresh plant leaves (500 mg) of each treatment were homogenized with 1.5 mL of TCA (tri-chloroacetic acid) (5% *w*/*v*) and centrifuged at 1200 rpm for 10 min. In 2.0 mL of diluted extract, 2 mL of thiobarbituric acid (TBA, 0.5% *w*/*v*) prepared in 1% butyl hydroxytoluene, 15% TCA, and 0.25 N HCl were mixed. The reaction solution was incubated for 30 min in a boiling water bath and immediately cooled to terminate the reaction. The cooled reaction mixtures were centrifuged at 10,000 rpm for 5 min, and the OD at 532 nm of the supernatant was taken. The MDA content was represented as nmol/g FW from the standard curve of MDA (range of 0.1–10 nmoL).

### 2.17. Quantitative Reverse Transcription-Polymerase Chain Reaction (qRT-PCR) of Stress-Related Gene Expression

To analyze the gene expression pattern of stress-related genes in sugarcane, qRT-PCR was performed. In brief, the total RNA from all treated sugarcane plant leaves was extracted by Trizol reagent (Invitrogen, Carlsbad, CA, USA), following the manufacturer’s instructions. Extracted RNA was spectrophotometrically quantified and reverse-transcribed into cDNA through PrimeScriptTM RT Reagent Kit (TaKaRa, Shiga, Japan, China), following the manufacturer’s instructions. Further RT-PCR was performed with a final 20 µL reaction mixture, containing 2 µL of 10-fold diluted first-strand cDNA, 10 pM of sugarcane stress-specific primers of *ScDREB2A*, *SuCAT*, and *SuSOD* (Supplementary Table S1), and 10 µL of SYBR Premix Ex TapTMII (Takara, Kyoto, Japan). All RT-qPCR reactions were performed in a real-time PCR system (Bio-Rad, Hercules, CA, USA) with the following programming: initial denaturation at 95 °C for 3 min, followed by 35 cycles of 95 °C for 15 s, 58 °C for 30 s, and 72 °C for 30 s, with a subsequent melting cycle from 60 to 95 °C. All RT-qPCR reactions were performed with reference gene GAPDH in three replicates. To compare relative expression, the delta–delta CT method was used.

### 2.18. Statistical Analysis

The present study’s data values obtained from different experiments are the mean ± standard error of three individual replications of the experiments. Through SPSS (ver. 16.0) statistical software (IBM, Armonk, NY, USA), all of the obtained data were analyzed using analysis of variance (ANOVA) and Duncan’s multiple range test (DMRT) at *p* ≤ 0.05.

## 3. Results

### 3.1. Isolation and Characterization of the Salt-Tolerant Rhizobacterial Strain

In the present study, using the salt enrichment technique, a fastidious and dominant bacterial colony designated as ASN-1 was obtained on an NA medium plate supplemented with 10% NaCl from the rhizosphere soil of sugarcane. The isolate ASN-1 was subjected to morphological, biochemical, and physiological characterization. Morphological observation showed that the strain ASN-1 was a Gram-positive rod that was cream-colored, circular, medium-sized, irregular, and flat. The biochemical characteristics revealed that ASN-1 was positive for catalase, oxidase, citrate utilization, methyl red, indole, phenylalanine deaminase, nitrate reduction, and lysine utilization but was negative in the Voges–Proskauer test, for ornithine utilization and for H_2_S production (Supplementary Table S2).

Further, the metabolic profile of the strain ASN-1 was characterized by using the “BIOLOG phenotype micro-array™ GNIII-carbon plate” with BIOLOG GNIII MicroPlate™. The results showed that ASN-1 utilized or was positive for 15 sugars, 2 hexose-PO_4_, 9 amino acids, 7 hexose acids, 2 reducing sugars, 7 carboxylic acids, esters, and fatty acids and was chemically sensitive to 13 substrates (Tables S3 and S4).

### 3.2. Intrinsic Salt Stress Tolerance Assay

**Figure 2:**
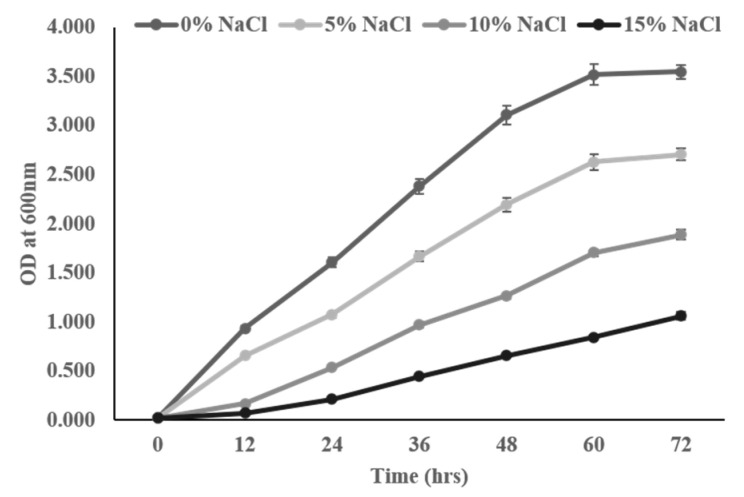
Growth kinetics of the salt-tolerant bacterial strain ASN-1 at different NaCl concentrations.

The growth of the selected bacterial isolate ASN-1 was studied at different salt (NaCl) stress levels (5, 10, 15 and 20%). The results showed that bacteria were able to grow in the presence of up to 15% NaCl. In addition, it was revealed that higher NaCl stress did not negatively affect bacterial growth; however, growth was sluggish for the first few hours with increasing concentrations of NaCl (Figure 2).

### 3.3. Molecular Identification and Phylogenetic Analysis

**Figure 3:**
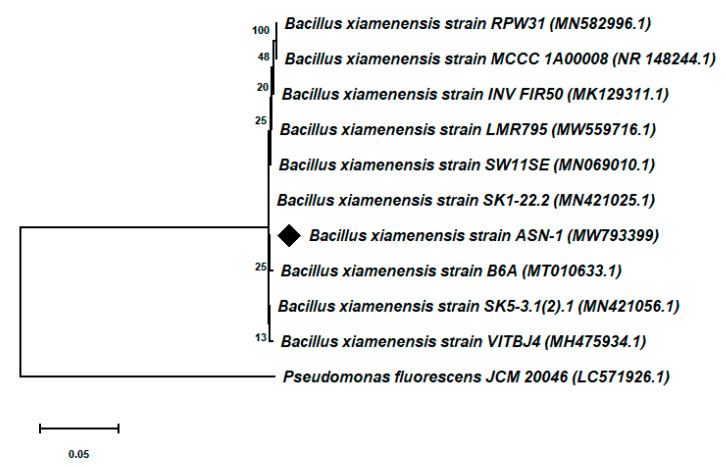
Phylogenetic neighbor-joining dendrogram on the basis of 16S rRNA sequences presenting relationships between the *Bacillus xiamenensis* strain ASN-1 and related taxa. *Pseudomonas fluorescens* JCM20046 was used as an out group.

To identify the potential salt-tolerant bacterial isolate ASN-1 at the molecular level, nearly 1.5 kb amplicons of the 16S rRNA gene of the bacteria were obtained through 16S rRNA gene sequencing analysis. Based on the BLAST search result against the available sequences in the NCBI Genbank database, the isolate ASN-1 showed 100% sequence similarity with *Bacillus xiamenensis.* Phylogenetic analysis of the isolate ASN-1 with similar sequence of NCBI database was conducted based on the neighbor-joining method with 1000 bootstrap sampling (Figure 3). The 16S rRNA gene sequence of the bacteria *Bacillus xiamenensis* ASN-1 was submitted to the NCBI Genbank under the accession number MW793399 (Figure 3).

### 3.4. Screening for Plant-Growth-Promoting Attributes

The results of plant-growth-promoting attributes showed that bacterial isolate ASN-1 was able to produce multiple PGP attributes. The amount of IAA production by the isolate ASN-1 was 196.48 ± 1.1 µg/mL (Table 1). Further, the clear zone around the bacterial colony growth on Pikovaskaya’s agar medium was observed, indicating the positive results for the solubilization of phosphates (Table 1). Similarly, positive results of the isolate ASN-1 were observed for ACC deaminase, siderophore, HCN, and ammonia production. Furthermore, the isolate ASN-1 was able to produce EPS. Interestingly, the isolate ASN-1 was able to retain all of the studied PGP functional traits as well as the EPS production capacity at 5% and 10% NaCl concentrations (Table 1).

### 3.5. Biofilm Formation

**Figure 4:**
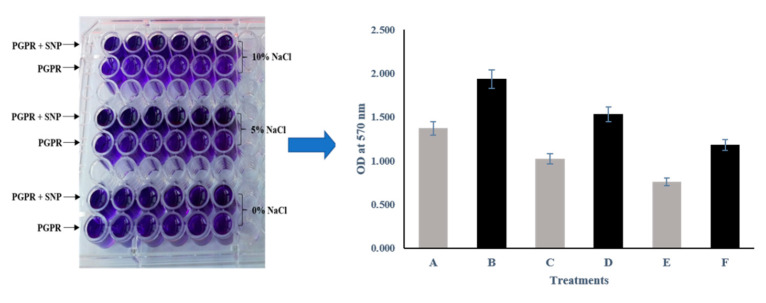
Effect of SNP (100 µM) amendment on PGPR biofilm formation under 5% and 10% NaCl stress. A: PGPR; B: PGPR + SNP; C: PGPR + 5% NaCl; D: PGPR + SNP + 5% NaCl; E: PGPR + 10% NaCl; F: PGPR + SNP + 10% NaCl.

The results of biofilm formation showed that SNP significantly increases the biofilm formation capacity of the strain ASN-1. Under unstressed conditions, SNP increases biofilm formation capacity 40.98% more than non-SNP treated culture. Under salt stress conditions, SNP treatment increased the biofilm formation by 49.9 and 37.4% at 5 and 10% NaCl concentrations, respectively, compared to the non-SNP-treated salt-stressed culture (Figure 4). Thus, the results indicate that SNP induced higher biofilm formation, strengthening effective root colonization.

### 3.6. Effect of PGPR Isolates ASN-1 and SNP (NO Donor) on the Mitigation of Salt Stress and the Growth Promotion of Sugarcane under Greenhouse Conditions

**Figure 5:**
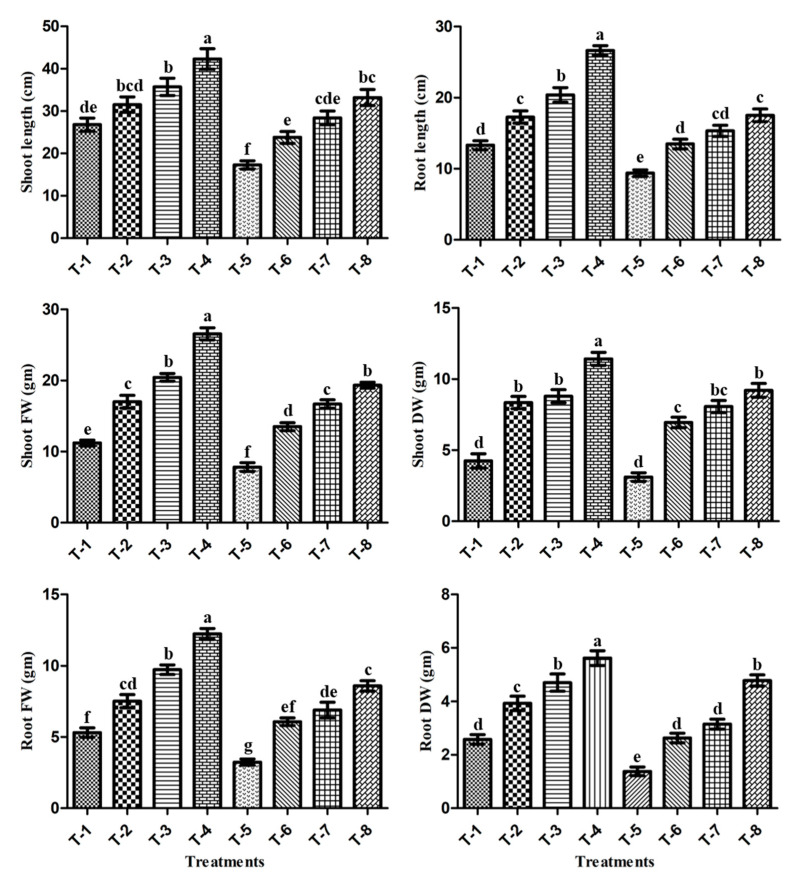
Effect of salt-tolerant PGPR ASN-1 and SNP on sugarcane growth parameters. T-1: Un-inoculated control; T-2: SNP; T-3: PGPR ASN-1; T-4: PGPR ASN-1 + SNP; T-5: NaCl (200 mM); T-6: NaCl + SNP; T-7: NaCl + PGPR ASN-1; T-8: NaCl + PGPR ASN-1 + SNP; FW: fresh weight; DW: dry weight. The mean ± SE, *n* = 3 is represented. The alphabet letters present on each data bar representing noteworthy variances (*p* < 0.05) after the DMRT test.

The pot experiment results showed that salt stress negatively affected all plant growth parameters (root-shoot length, fresh weight, and dry weight) (Figure 5). The results showed that salt stress (T-5) decreased the shoot length by 35.5% and the root length by 29.4%, as compared to non-stressed, un-inoculated control plants (T-1). However, the application of PGPR isolates ASN-1 and SNP, either alone or in combination, alleviates the negative effect of salt stress and enhances the shoot and root length over un-inoculated plants grown in salt stress conditions (Figure 5). The maximum enhancement of shoot length (92.4%) and root length (86.7%), in relation to un-inoculated salt-stressed sugarcane plants (T-5) was observed when PGPR and SNP were applied in combination (T-8), followed by PGPR alone, i.e., T-7 (65 and 63.5%), and SNP alone as T-6 (37.8 and 43.8%) (Figure 5). Furthermore, salt stress (T-5) decreased the fresh shoot weight (SFW) and shoot dry weight (SDW) by 30.1% and 26.8%, respectively, as compared to non-stressed plants in T-1 (Figure 5), whereas the collective application of PGPR isolates ASN-1 + SNP (T-8) significantly enhanced the SFW and SDW by 147.3% and 196% in relation to un-inoculated salt-stressed plants (T-5), more so than PGPR ASN-1 alone (T-7) (113.4 and 159.4%) and SNP alone (T-6) (72.7 and 123.4%) (Figure 5). Furthermore, salt stress (T-5) also inhibited the fresh root weight (RFW) by 39.1% and root dry weight (RDW) by 46.5%, as compared to T-1 non-stressed un-inoculated control plants, whereas the combined application of PGPR isolates ASN-1 + SNP (T-8) manifested a significant increase in RFW by 165.8% and in RDW by 247.3%. Additionally, apart from T-8 treatment, these values were also increased by a single application of PGPR (T-7) and SNP (T-6), respectively, compared to salt-stressed plants (T-5) (Figure 5). The overall results of the pot experiment showed that, compared to a single inoculation of the PGPR isolate ASN-1 (T-7) and SNP (T-6), the combined use of PGPR + SNP (T-8) was found to better mitigate salt stress and better promote the growth of sugarcane plants under greenhouse conditions.

### 3.7. RWC and Electrolytic Leakage (EL)

Concomitant with growth parameters, salt stress negatively affected the RWC and electrolytic leakage of the sugarcane plants. Compared to non-stressed un-inoculated sugarcane plants (T-1), salt stress (T-5) significantly decreased the RWC by 42.1% (Table 2). However, sugarcane plants treated with the PGPR and SNP, alone or in combination, had significant positive effects on RWC under normal non-stressed and salt-stressed conditions. The results showed that a significant improvement in RWC, by 91.8%, was observed in the collective application of PGPR ASN-1 and SNP (T-8) in sugarcane plants, higher than single inoculations of PGPR isolate ASN-1 (70.2%) and SNP (56.7%) (Table 2).

Furthermore, the percentage of electrolytic leakage significantly increased by 54.6% in sugarcane plants under salt stress (T-5), as compared to the non-stressed control plants (T-1) (Table 2), while the application of PGPR and SNP significantly reduced the ionic discharge under salt stress conditions (Table 2). Among all tested treatments, the highest reductions (47.2%) in electrolyte leakage were obtained in plants treated with both PGPR isolate ASN-1 and SNP (T-8), higher than PGPR treatment alone, i.e., T-7 (38.8%), and SNP treatment alone, i.e., T-6 (30.4%), under salt stress conditions (Table 2).

### 3.8. STI Index

Similar to the RWC and EL results, a considerable improvement in STI was observed in sugarcane plants treated with the PGPR strain and SNP (Table 2). The results showed that the lowest STI value (0.7) was observed in positive salt control (T-5) sugarcane plants, while the highest STI value (3.1) was recorded in plants treated with PGPR ASN-1 + SNP (T-8), i.e., higher than plants treated with the PGPR strain ASN-1 alone (2.5) and SNP alone (2.1). These findings suggest that inoculating sugarcane plants with PGPR, either with or without SNP fertilizer, improves salinity tolerance.

### 3.9. Analysis of Ionic Content

Results of ionic accumulation showed that salinity (T-5) remarkably increased the Na^+^ ion by 207.7% and decreased the K^+^ ion concentration by 39.1% in the root of un-inoculated sugarcane plants (Table 2). This ionic imbalance disrupts the plant cell membranes and causes leakage of the electrolyte. However, PGPR isolates ASN-12 and SNP fertilization alleviate the deleterious effect of salt stress on plant growth by significantly regulating the accumulation of Na^+^ and K^+^ ions in the sugarcane plants grown under non-stressed and salt-stressed conditions. The results showed that, under salt stress conditions, the combined effect of PGPR isolates ASN-1 + SNP (T-8) led to a significant decrease in Na^+^ ion accumulation (50.8%) and increased K^+^ ion accumulation (230.2%) in sugarcane plants, followed by decrease in Na^+^ and an increase in K^+^ ions in the T-7 (PGPR isolate ASN-1) and T-6 (SNP) treatments, respectively (Table 2).

### 3.10. Chlorophyll Content (SPAD Value)

**Figure 6:**
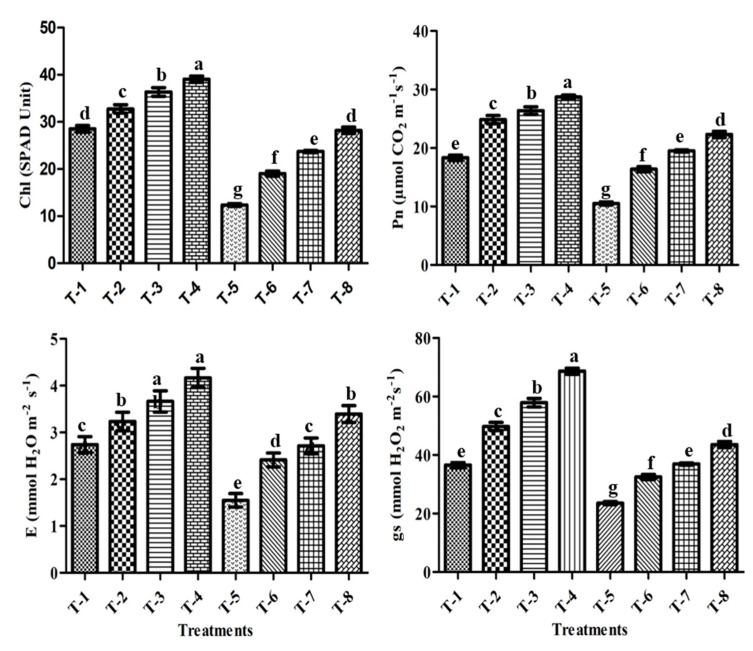
Effect of salt-tolerant PGPR ASN-1 and SNP on sugarcane photosynthesis attributes. T-1: Un-inoculated control; T-2: SNP; T-3: PGPR ASN-1; T-4: PGPR ASN-1 + SNP; T-5: NaCl (200 mM); T-6: NaCl + SNP; T-7: NaCl + PGPR ASN-1; T-8: NaCl + PGPR ASN-1 + SNP. Chl: chlorophyll; *Pn*: net photosynthesis rate; *gs:* stomata conductance; *E:* transpiration rate. The mean ± SE, *n* = 3 is represented. Letters on each column bar represent significant variances (*p* < 0.05) after the DMRT test.

The leaf chlorophyll content (SPAD value) of all the treated sugarcane plants varied under normal and salt stress conditions. Under normal conditions, the SPAD value of sugarcane treated with a combined application of PGPR ASN-1 and SNP increased by 36.9%, more so than PGPR alone (27.4%) and SNP alone (14.8%) (Figure 6). The results showed that salt stress negatively affected the SPAD value of the sugarcane plants and decreased by 56.7% compared to non-stressed control plants (T-1). Although salt stress reduced the chlorophyll content, plants treated with a combined application of PGPR ASN-1 and SNP performed better and significantly enhanced the leaf greenness SPAD value of sugarcane plants by 128.3% compared with un-inoculated stressed control plants (T-5), more so than single inoculations of PGPR and SNP (Figure 6). The results showed that, under both stressed and unstressed sugarcane plants, the measured SPAD value of photosynthetic pigments in plants treated with PGPR + SNP (T-8) was higher than in single inoculations of PGPR and SNP.

### 3.11. Gas-Exchange Parameters

No significant differences in gas-exchange values were identified between plants with and without inoculation in the absence of NaCl. However, salt stress had adverse effects on all parameters (Figure 6).

In the present study, salt stress resulted in a significant decrease in the net photosynthetic rate (Pn) (42.5%) compared to non-stressed un-inoculated sugarcane plants (Figure 6). However, the plants treated with PGPR and SNP exhibited a significant increase in Pn compared to plants with salt stress (T-5). The results showed that the combined application of PGPR + SNP increased the maximum Pn by 111.9%, higher than the applications of PGPR alone (T-7) (85.1%) and SNP alone (T-6) (55.4%) (Figure 6).

Similarly, due to salt stress, the stomatal conductance (gs) value was decreased by 35.5% compared with non-stressed un-inoculated control plants (T-1) (Figure 6). However, PGPR and SNP plants displayed enhancements in gs under salt-stressed conditions, more than in plants without a PGPR application. Again, the combined application of PGPR + SNP (T-8) significantly increased the gs by 84.7%, compared to the un-inoculated salt-stressed sugarcane plants (T-5), more so than PGPR alone (T-7) and SNP alone (T-6) (Figure 6).

A similar trend was observed in the case of the transpiration rate (E), such as emerged with Pn and gs, where salt stress treatment (T-5) decreased the E value by 43.3% in comparison with the un-inoculated non-stressed plants (T-1) (Figure 6). The plants with combined PGPR + SNP treatment maintained a higher E by 118.8% in comparison with the un-inoculated salt-stressed plants, higher than the single treatments of PGPR (T-7) (74.8%) and SNP (T-6) (55.4%) (Figure 6).

### 3.12. Proline and TSS Contents

The observed effects of different treatments of PGPR and SNP on proline, TSS, and protein content under salt stress conditions are shown in Table 2. Salt stress increased proline content by 71.4% in sugarcane plants compared to the T-1 non-stressed un-inoculated control plants. However, a more prominent effect was revealed with the application of PGPR and SNP. Among all treatments, the combined application of PGPR + SNP (T-8) significantly enhanced the proline content by 101.5%, more so than the single treatments of PGPR (T-7) and SNP (T-6) (Table 2).

In the case of TSS, salt stress (T-5) significantly reduced the TSS level by 54.3% compared to T-1. However, similar to proline observations, PGPR and SNP together had a significant effect on the accumulation of TSS in the sugarcane plants under salt stress conditions. The results showed that salt-stressed sugarcane plants treated with PGPR and SNP together (T-8) showed a significantly increased level of TSS content, i.e., 39.9%, higher than the increase in the T-7 (single PGPR) (20.4%) and in T-6 (single SNP) (14.7%) treatments (Table 2).

### 3.13. Antioxidants Enzyme Activities

**Figure 7:**
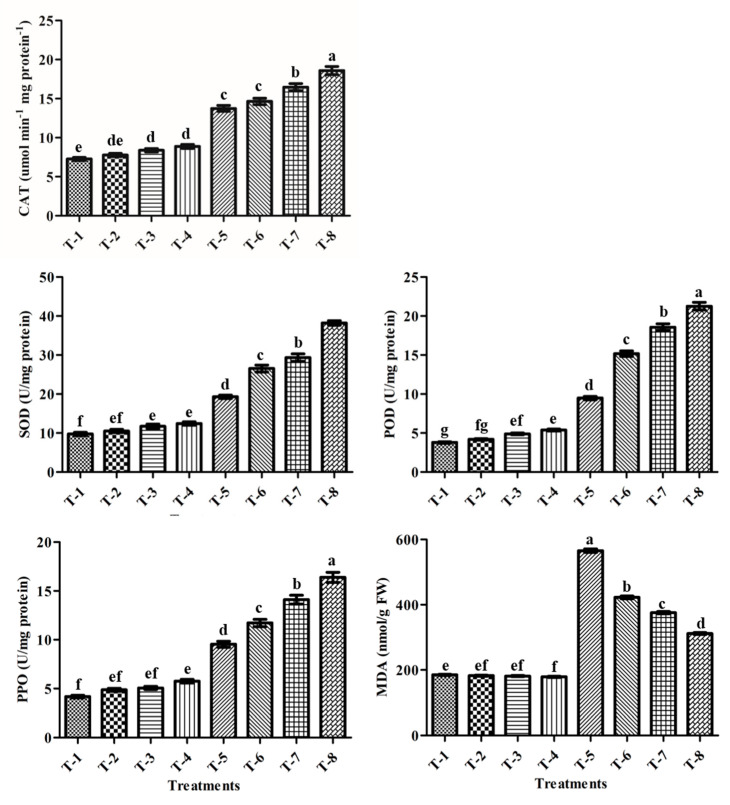
Effect of salt-tolerant PGPR ASN-1 and SNP on antioxidant enzyme activity. T-1: Un-inoculated control; T-2: SNP; T-3: PGPR ASN-1; T-4: PGPR ASN-1 + SNP; T-5: NaCl (200 mM); T-6: NaCl + SNP; T-7: NaCl + PGPR ASN-1; T-8: NaCl + PGPR ASN-1 + SNP. The mean ± SE, *n* = 3 is represented. Letters on each bar represent significant variances (*p* < 0.05) after the DMRT test.

To understand the scavenging mechanism of ROS-induced oxidative stress, the antioxidant enzyme activities (CAT, SOD, PPO, and POD) of all the treated sugarcane plants were evaluated under non-stress and salt stress conditions. The results of the antioxidant enzyme test revealed that all treatments had a significant impact on the antioxidant enzyme activities of sugarcane plants under salt stress (Figure 7). Salt stress treatment (T-5) showed levels of CAT, SOD, PPO, and POD were enhanced by 89%, 98%, 128.7%, and 150.4%, respectively, compared to non-stressed control plants (T-1) (Figure 7). Furthermore, these antioxidant enzyme activities were significantly improved by the application of salt-tolerant PGPR isolate ASN-1 and SNP. The results showed that the combined use of PGPR + SNP (T-8) enhanced the CAT by 35.3%, the SOD by 98.1%, the PPO by 71.8%, and the POD by 124.1% compared to the un-inoculated salt-stressed plants (T-5) (Figure 7). Under normal conditions, there were no significant variations in the activity of the three enzymes between treatments and the relevant controls (Figure 7). These results revealed that T-8 (PGPR ASN-1 in association with SNP) was more effective in improving the activities of enzymes. SNP alone (T-6) was also effective in boosting antioxidant enzyme activities; however, the increment was lower than PGPR inoculation alone (T-7).

### 3.14. MDA Contents

Salt stress (T-5) resulted in an increased level of lipid peroxidation in terms of a higher production of malondialdehyde (MDA), which was increased by 205.5% over un-inoculated non-stressed plants (T-1) (Figure 7). However, plants with PGPR and SNP treatments alleviated the stress-induced lipid peroxidation by significantly reducing MDA content, as compared to un-inoculated sugarcane plants grown with salt stress. The results showed a maximum reduction of 44.8% in the combined use of PGPR isolate ASN-1 + SNP (T-8) compared to un-inoculated salt stress plants (T-5), a greater reduction than the single applications of PGPR (T-7: 33.5%) and SNP (T-6: 25.2%) (Figure 7).

### 3.15. qRT-PCR Gene Expression Analysis

**Figure 8:**
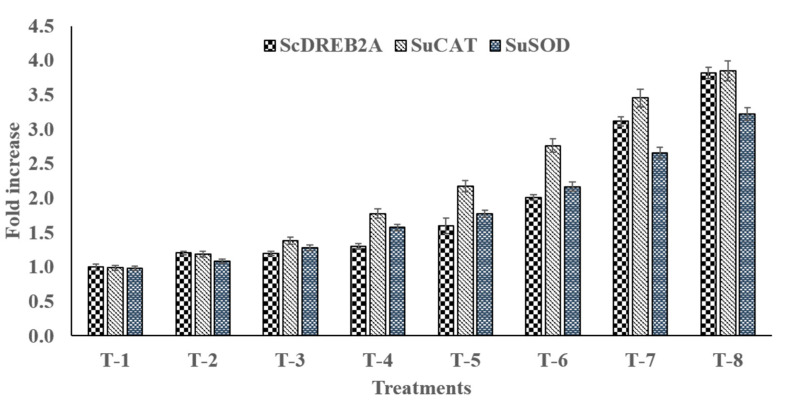
Effect of salt-tolerant PGPR ASN-1 and SNP application on expression of *ScDREB2A*, *SuCAT* and *SuSOD* gene. T-1: Un-inoculated control; T-2: SNP; T-3: PGPR ASN-1; T-4: PGPR ASN-1 + SNP; T-5: NaCl (200 mM); T-6: NaCl + SNP; T-7: NaCl + PGPR ASN-1; T-8: NaCl + PGPR ASN-1 + SNP. The mean ± SE, *n* = 3 is represented. Letters on each bar represent significant variances (*p* ≤ 0.05) after the DMRT test.

To investigate the stress tolerance mechanism, expression analysis of one dehydration responsive element binding protein (*ScDREB2A*) gene and two defense-related antioxidant enzymes genes (*SuCAT* and *SuSOD*) was performed using real-time qRT-PCR (Figure 8). Variability in the expression pattern of all three tested genes was observed in un-inoculated and inoculated salt-stressed plants. RT-PCR analysis demonstrated that, compared to unstressed plants, the expression of the *ScDREB2A* gene was upregulated 1.6-fold in salt-stressed plants (T-5), whereas plants treated with PGPR and SNP further enhanced expression of the *ScDREB2A* gene. The results showed that the combined application of PGPR and SNP (T-8) (*p* ≤ 0.05) repressed the expression of the *ScDREB2A* gene 3.8-fold, which was higher than the repression observed in plants treated with PGPR alone (3.1-fold) and SNP alone (2.0-fold) (Figure 7). Furthermore, the expression of *SuCAT* and *SuSOD* genes was higher by 2.2- and 1.8-fold in salt-stressed un-inoculated sugarcane plants. Moreover, combined PGPR ASN-1 and SNP treatment further increased *SuCAT* and *SuSOD* gene expression by 3.9- and 3.2-fold, respectively, upon salt stress, more than PGPR alone (3.5- and 2.7-fold) and SNP alone (2.8- and 2.2-fold). The results showed that, under normal non-stressed conditions, the application of PGPR and SNP remarkably regulated the expression of these stress-tolerant genes in sugarcane plants. On the basis of RT-PCR gene expression analysis, it can be concluded that PGPR and SNP regulate the expression of stress-responsive genes and can be applied to enhance tolerance to salt stress in sugarcane plants.

### 3.16. Heatmap of Pearson’s Correlation Analysis

**Figure 9:**
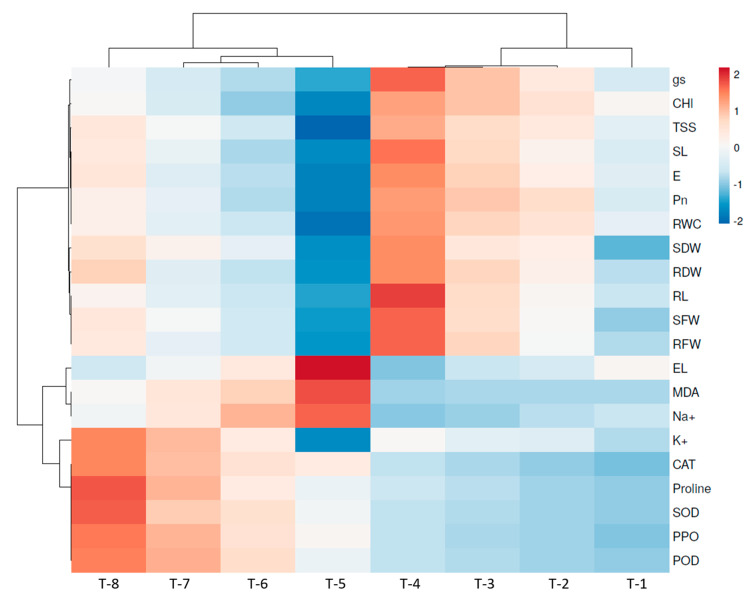
Heatmap multivariate data analysis showing the responses of different variables of plants against the different treatments applied. T-1: Un-inoculated control; T-2: SNP; T-3: PGPR ASN-1; T-4: PGPR ASN-1 + SNP; T-5: NaCl (200 mM); T-6: NaCl + SNP; T-7: NaCl + PGPR ASN-1; T-8: NaCl + PGPR ASN-1 + SNP. SL: shoot length; RL: root length; SFW: shoot fresh weight; SDW: shoot dry weight; RFW: root fresh weight; RDW: root dry weight; Chl: chlorophyll; Pn: net photosynthesis rate; gs: stomata conductance; E: transpiration rate; RWC: relative water content, EL: electrolytes leakage; TSS: total soluble sugar; CAT: catalase; SOD: superoxide dismutase; POD: peroxidase; PPO: polyphenol oxidase; MDA: malondialdehyde.

Heatmap analysis showed that the plant growth variables exhibited differential responses in the integrated heatmap against the different treatments (Figure 9). The multivariance heatmap analysis clearly suggests that salinity stress imposed negative effects on all of the parameters of the plants, while plants treated with halotolerant PGPR and SNP influenced the vegetative, physiological, and biochemical parameters as well as the antioxidant enzyme expression of the sugarcane plants (Figure 9).

### 3.17. Root Colonization of PGPR Isolates ASN-1

**Figure 10:**
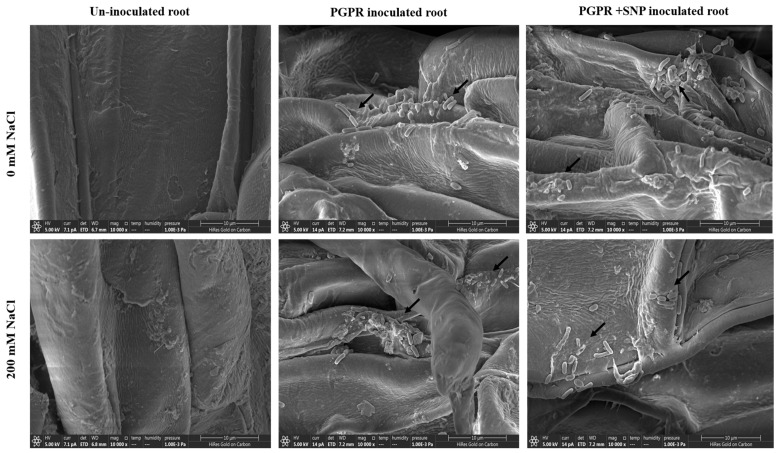
Scanning electron microscopic image showing root colonization and biofilm formation of salt-tolerant PGPR strain ASN-1 under NaCl and SNP modifications.

The root colonization capability of the PGPR isolate ASN-1 was estimated by scanning electron microscopy (SEM) in inoculated sugarcane plant root in comparison with un-inoculated non-stressed (T-1) and NaCl-stressed plants (T-5). SEM analysis showed an abundance of PGPR isolate ASN-1 cells on the surface and in crevices of the root with or without SNP in inoculated salt-stressed and non-stressed sugarcane plants (Figure 10).

## 4. Discussion

Among the various approaches adopted to further sustainable sugarcane productivity and to maintain an adequate food supply under the harmful effects of soil salinity, the use of halotolerant root colonizing PGPR and the exogenous application of NO are important components that may mitigate salinity stress and enhance plant growth [28,66,67]. It is an economically feasible and cost-effective approach that induces a systemically acquired tolerance in plants against salt tolerance and, overall, leads to improved plant growth. Given the importance of PGPR and NO in the mitigation of plant salt stress and growth promotion, the goal of the present study was intended to evaluate the influences of halotolerant PGPR and the exogenous use of SNP as a NO donor in alleviating the deleterious harmful effects of salt stress on sugarcane plant growth and development by looking into a variety of physiological, biochemical and molecular factors that affect salt tolerance. Taking this into account, the sugarcane-associated, salt-tolerant rhizosphere bacteria ASN-1 was isolated through an NaCl enrichment technique [68]. The 16S rRNA gene sequencing and phylogenetic analysis confirmed the taxonomic position of the rhizobacteria as *Bacillus xiamenensis* (NCBI Accession No. MW793399) (Figure 3). Our findings revealed that the strain ASN-1 can have a NaCl concentration of up to 15% (Figure 2) and exhibited varying levels of multiple PGP traits, such as the production of IAA, phosphate solubilization, ACC deaminase activity, and the production of siderophores, HCN, ammonia, and EPS under normal salt-stressed conditions, which indicated its potential to express relevant PGP traits for plant growth promotion in the saline soil–plant system (Table 1). Several earlier studies reported that, due to its endospore-producing capacity, *Bacillus* spp. has been well known as a genus for stress tolerance and plant growth promotion [69,70]. However, a synergistic study of *Bacillus* spp. with NO for the mitigation of salt stress and plant growth promotion has rarely been performed [67,71].

Therefore, a pot experiment was carried out to assess the efficiency of the selected PGPR strain ASN-1 and exogenously applied NO to ameliorate salt stress and enhance sugarcane plant growth in connection to several growth-related parameters. The results of the pot experiment showed that, being a stress-sensitive crop, salinity stress disrupts the sugarcane metabolism and biological activities, ultimately reducing overall sugarcane plant growth and biomass (Table 2, Figure 5) [72,73]. However, plants treated with PGPR isolates ASN-1 with SNP significantly ameliorated salt stress and improved the sugarcane plant growth and dry biomass under salinity stress. The results of the present study indicate that the selected halotolerant PGPR isolate ASN-1 and SNP can significantly alleviate the salt-induced debilitating effects on plants and enhance the vegetative growth of sugarcane plants. Our results are in line with the study by Vaishnav et al. [66], where PGPR and SNP application significantly enhanced soybean growth under salt stress conditions. Several studies have estimated that improved indole acetic acid (IAA), ACC deaminase, siderophore, and ammonia production as well as the mineral solubilizing activity of the applied PGPR may work together in a coordinated network to help plants to stimulate the growth of a variety of crops under salt stress (Table 1) [74,75]. Among the various attributes, phytohormone IAA synthesized by the PGPR can improve the root system’s architecture, which consequently allows plants to take in additional water and nutrients [76]. On the contrary, the activity of ACC deaminase of bacterial strains might reduce stress ethylene production by breaking down ACC (a direct precursor of ethylene biosynthetic) into ammonia and α-ketobutyrate, thereby increasing salinity tolerance in the plant [77]. PGPR can also enhance the availability of essential nutrient phosphorus (P) to plants by the secretion of enzyme phosphatase, chelating substances, or organic acids that support decreases in the pH of the rhizosphere [78]. In line with the present study’s findings, several earlier reports have also established the capability of PGPR to stimulate the growth of a variety of crops under salt stress [75,79,80]. Our results are also in accordance with the earlier studies of Sarropoulou et al. [81] and Chen et al. [82], who reported that the application of SNP under salt stress conditions enhanced the growth characteristics of plants by relaxing the cell wall, increasing membrane fluidness and osmotic pressure, inducing cell enlargements, and regulating the antioxidant enzyme system.

Relative leaf water (RWC) contents represent the status of water in plants; additionally, a reduction in the RWC of plants due to lower osmotic potential and the loss of cell turgidity are generally the first noteworthy effects of osmotic stress [83,84]. Thus, keeping RWC in good shape in cells and tissues allows metabolic activity to be sustained by osmotic adjustments and other salinity adaptations [85]. The present study showed that the application of PGPR and SNP reduced the negative effect of salt stress on the RWC of sugarcane plants (Table 2). The results obtained are in accordance with several scientists who have previously documented a significant rise in RWC in wheat, mung bean, chickpea, and mustard plants treated with PGPR and NO under saline circumstances [82,86,87].

Higher salt concentration leads to ion disproportion in the cytosol of plants. Due to this factor, an increased level of Na^+^ ions and a reduced level of K^+^ ions was observed, which caused ionic toxicity and reduced plant development [88]. In this study, un-inoculated sugarcane plants under salt stress exhibited an increased amount of Na^+^ ion and a decrease in K^+^ ion content. However, plants inoculated with halotolerant strain ASN-1 and SNP, either alone or in combination, lowered the Na^+^ ion and increased the K^+^ ion accumulation and thus increased the STI level of the plants (Table 2). The greater EPS production of PGPR could be the reason for the limited Na^+^ ion absorption in plants because the EPS matrix can gripe the cations (Na^+^) and form an extensive biofilm by modifying the root structure and boosting the expression of K^+^ ion affinity transporters, whereas NO protects plants from salt stress by enhancing the plasma membrane (PM) H^+−^ATPase development, which is essential for balancing the K^+^/Na^+^ ion ratio [89]. These findings are similar to several previous studies, where researchers reported that PGPR and NO mitigate salt stress in plants by the efflux of the Na^+^ ion and the influx of the K^+^ ion in the plants [2,89,90].

Under salinity stress, due to the destruction of the photosynthetic apparatus, plants displayed negative signs, such as leaf yellowing, wilting, and withering. These morphological alterations in the leaves are linked to plant metabolic disorders, such as decreased photosynthesis and chloroplast activity and increased respiration, thus finally reducing the chlorophyll content [90,91]. However, in the present study, PGPR and SNP application exhibited a higher level of chlorophyll pigments in salinity-stressed sugarcane plants (Figure 6). Similar to our results, earlier studies have been published where the application of PGPR and NO prevented intercostal chlorosis in leaves and provoked the enhancement of chlorophyll content in various crops under saline conditions [84,92,93].

Leaf gas exchange attributes, such as the photosynthesis rate, transpiration rate, stomatal conductance, net assimilation rate, and CO_2_ concentration, recognized as plant stress indicators, are important for plant growth and development [94]. All of these physiological processes are interlinked with each other and are active in parallel under salt stress conditions. Any activity disruption of one process will result in the decreased efficiency of the others. In the present study, the sugarcane plant showed significant reductions in gas exchange attributes, such as the photosynthetic rate, stomatal conductance, and transpiration rate, under salinity stress conditions (T-5) (Figure 6), which could have hindered cell growth and development. Membrane instability, cell turgor fluctuation, reduced stomatal conductance, and nutritional imbalance may reduce gas exchange parameters in salinized conditions [95]. However, in the present study, PGPR- and SNP-inoculated plants had remarkably maintained and upregulated the photosynthetic rate, stomatal conductance, transpiration, and intercellular CO_2_ concentration in plant leaves in comparison with the plants without any inoculation, which demonstrates sustained plant growth as well as health (Figure 6). These results support the findings of Kanwal et al. [96], Yasin et al. [97], Yasmeen et al. [98], and Ansari et al. [99], who detected enhancements in gas exchange traits with PGPR-treated black gram, maize, and wheat plants under salinity stress. In addition, Cechin et al. [100] and Wani et al. [101] reported that SNP as a NO donor also sustains the gas exchange parameters under abiotic stress conditions.

Furthermore, the osmolyte proline and TSS are the major biochemical markers for salt stress tolerance in plants [102]. Under salinity stress conditions, proline accumulation in the cytosol assists osmotic adjustment in the cytoplasm and maintains sub-cellular components of the plant cell, such as cell membranes, macro molecule, buffering cellular redox potential, and scavenging free radicals, under salinity stress [103]. The current study showed that proline levels were considerably higher in sugarcane plants treated with PGPR and SNP, indicating a lower effect of stress severity on salt stress plants. This demonstrated the vital role of proline in protecting plants against the stressful impact of salt. In addition to proline, soluble sugars were also found higher in the combined application, which preserves the structure of macromolecules and membranes, and functions as signals that control various processes associated with plant growth and development in extreme environments [104].

In the present study, an increase in MDA content of sugarcane plants was observed under salt stress conditions, which may be due to the lipid peroxidation in the plant cell membrane, and an increase in cell membrane permeability, resulting in the enhanced leakage of electrolytes from the cells [75,105]. Increased MDA levels may cause various developmental signals such as chlorophyll loss, increased cell membrane permeability and electrolyte leakage, macromolecule breakdown, significant nutrient remobilization, and early senescence, resulting in a shorter crop development time. However, PGPR and SNP fertilization demonstrated notably low MDA content and electrolyte leakage, due to which there was probably less damage to the plant’s cell membrane (Figure 7) [106,107]. Our results are inconsistent with those of Vaishnav et al. [66] and Ilyas et al. [86], who reported that PGPR and SNP fertilization demonstrated notably low electrolyte leakage and that, as a result, there is less cell membrane damage to the plants.

It is well documented that, in plants, the ROS-scavenging antioxidative enzyme defense systems (such as CAT, SOD, PPO, POD, and APX) efficiently compensate for the stress-induced oxidative damage [108,109]. Under salt stress conditions, these antioxidative enzymes’ upregulation could help protect plants from oxidative injury [110,111]. The results of the current study presented that the antioxidant enzymatic activities were significantly increased through PGPR and SNP applications in sugarcane plants (Figure 7). These findings are corroborated by observations in earlier studies wherein, following PGPR inoculation, antioxidant enzymes were upregulated to counteract salt stress, resulting in the increased removal of harmful ROS molecules [111,112,113]. Furthermore, the results of the present study are also in line with the studies of Agami et al. [114] and Pissolato et al. [115], who reported that the elimination of the superoxide (O_2_•^−^) and the strengthening of the antioxidant system in sugarcane plants under abiotic stress had been ascribed to the protective activity of exogenous NO donors. These results suggest that the antioxidative enzyme requirement by the plants could uphold the accumulation of ROS and protect against stress-induced oxidative injury.

Consistent with the accumulation of the antioxidant enzyme in sugarcane plants, the application of PGPR and SNP also stimulates the distinct upregulation of stress-related gene transcripts, such as *ScDREB2A*, *SuCAT*, and *SuSOD*, which may confer the salt stress tolerance of the host plant. The DREB genes were the first transcription factors to be linked to gene regulation in response to abiotic stressors. The overexpression of *DREB2A* during high salinity and drought stress suggests that the DREB protein plays a key role in dehydration-responsive gene expression [116,117]. In this study, PGPR and SNP play a key role in the overexpression of *DREB2A*, which may help the host plant rapidly resist stress (Figure 8). The observations of the present study are in line with Augustine et al. [118], who reported that the overexpression of the *Erianthus arundinaceus DREB2* gene augmented salinity and drought stress in sugarcane. Similarly, the result suggested that in sugarcane plants treated with PGPR and SNP, the enhancement of the mRNA expression level of antioxidant-enzyme-related genes CAT and SOD might enhance these enzyme’s activities (Figure 8). As a result, the cells are better protected against salinity-induced oxidative stress [119]. Therefore, it is plausible to conclude that PGPR and SNP may increase the expression of *ScDREB2A*, *SuCAT*, and *SuSOD* genes, which leads to the enhancement of better salt tolerance in sugarcane; these results were confirmed by the higher RWC, photosynthetic pigments, gas exchanger parameters, the accumulation of osmoprotectants, and antioxidant enzymes, without causing a reduction in biomass accumulation.

## 5. Conclusions

Our findings showed that the salt-tolerant PGPR strain ASN-1 and SNP supplementation as a NO donor effectively protected the sugarcane against the adverse effects of salt stress by managing the physiological, biochemical, and photosynthetic traits, and by the accumulation of compatible solutes and antioxidant enzyme activities. The results showed that under both, high levels of soil salinity, as well as normal conditions, the combined application of *Bacillus xiamenensis* ASN-1 and SNP was more successful than their individual usage. Therefore, in the era of climatic changes, the application of PGPR isolate ASN-1 and SNP could be a promising approach for salt stress management. This information can be used to drive research efforts aimed at enhancing abiotic stress tolerance and yielding sustainability in agriculturally important crops.

## Figures and Tables

**Table 1:** Plant growth promoting attributes of strain ASN-1.

Salinity Level (% NaCl)	IAA Production (μg/mL)	ACC Deaminase	P-Solubilization Index (PSI)	Siderophore Production	HCN Production	Ammonia Production	EPS Production
0%	196.48 ± 1.12	+++	+++	+++	++	+++	+++
5%	122.13 ± 1.52	++	++	++	++	++	+++
10%	74.62 ± 1.70	++	+	+	+	++	++

(+) production at a normal level (++) production at a medium level, (+++) production at a high level. Values are expressed as mean ± SE.

**Table 2:** Effect of PGPR and SNP on RWC, EL, ion content, proline, sugar content, and STI.

Treatment	RWC (%)	EL (%)	Na^+^ (mg/g DW)	K^+^ (mg/g DW)	Proline (mg/g FW)	TSS (mg/g FW)	STI
T-1	63.30 ± 1.19 ^d^	39.41 ± 0.74 ^c^	4.12 ± 0.14 ^e^	8.10 ± 0.24 ^f^	10.62 ± 0.44 ^f^	123.28 ± 1.17 ^e^	-
T-2	75.17 ± 1.43 ^b^	32.73 ± 0.61 ^d^	3.85 ± 0.13 ^e^	9.72 ± 0.25 ^e^	11.54 ± 0.48 ^f^	136.16 ± 0.90 ^c^	-
T-3	79.13 ± 1.50 ^b^	31.05 ± 0.58 ^d^	3.23 ± 0.10 ^f^	10.05 ± 0.12 ^e^	12.62 ± 0.52 ^ef^	141.41 ± 0.52 ^b^	-
T-4	88.03 ± 1.67 ^a^	27.24 ± 0.51 ^e^	2.84 ± 0.11 ^f^	11.19 ± 0.17 ^d^	13.80 ± 0.58 ^e^	150.19 ± 0.88 ^a^	-
T-5	36.60 ± 0.68 ^f^	60.95 ± 1.14 ^a^	12.69 ± 0.25 ^a^	4.93 ± 0.13 ^g^	18.19 ± 0.42 ^d^	90.32 ± 1.04 ^g^	0.7 ± 0.04 ^d^
T-6	57.37 ± 1.09 ^e^	42.38 ± 0.79 ^b^	10.41 ± 0.21 ^b^	12.39 ± 0.14 ^c^	23.39 ± 0.51 ^c^	118.36 ± 0.84 ^f^	2.1 ± 0.12 ^c^
T-7	62.30 ± 1.19 ^d^	37.25 ± 0.70 ^c^	8.12 ± 0.16 ^c^	14.83 ± 0.17 ^b^	30.20 ± 0.65 ^b^	129.29 ± 0.89 ^d^	2.5 ± 0.14 ^b^
T-8	70.23 ± 1.33 ^c^	32.13 ± 0.60 ^d^	6.23 ± 0.12 ^d^	16.28 ±0.18 ^a^	36.65 ± 0.91 ^a^	136.40 ± 0.96 ^c^	3.1 ± 0.18 ^a^

T-1: Un-inoculated control; T-2: SNP; T-3: PGPR ASN-1; T-4: PGPR ASN-1 + SNP; T-5: NaCl (200 mM); T-6: NaCl + SNP; T-7: NaCl + PGPR ASN-1; T-8: NaCl + PGPR ASN-1 + SNP; RWC: relative water content, EL: electrolytes leakage; TSS: total soluble sugar; STI: salt tolerance index. The mean ± SE, *n* = 3 is represented. Different letters on each bar indicate significant differences (*p* = 0.05) in bacterial growth after DMRT test.

## Data Availability

Not applicable.

## Supplementary Materials

The following are available online at https://www.mdpi.com/article/10.3390/microorganisms9112203/s1, Table S1: Morphological and biochemical characteristics of strain ASN-1; Table S2: Carbon source utilized by strain ASN-1; Table S3: qRT-PCR primers used in this study. Table S4: Substrate present in each well of Biolog Micro-Plate.
