# Supplementary material for: Insights into the Bacterial and Nitric Oxide-Induced Salt Tolerance in Sugarcane and Their Growth-Promoting Abilities
Source: Microorganisms. 2021 Oct 22;9(11):2203. doi: 10.3390/microorganisms9112203 (PMC8623439; doi:10.3390/microorganisms9112203)
Supplement: Supplementary file 1 [file microorganisms-09-02203-s001.zip › microorganisms-1376723-supplementary.pdf]

Supplementary Table S1. qRT-PCR primer used in this study

Gene name	Primer	Sequence (5'-3')	References
ScGAPDH	GAPDH-F1	CTCTGCCCCAAGCAAAGATG	Singh et al. [2019] [1]
	GAPDH-R1	TGTTGTGCAGCTAGCATTG	
SuDREB	ScDREB2A-F	CAGTGTTGCCAACGGTTCAT	Reis et al. [2014] [2]
	ScDREB2A-R	GTAGCGGATCAAAACCACTTTGT	
SuCAT	SuCAT-F	CTTGTCTGGAGCACATACACTTGGA	Chen et al. [2012] [3]
	SuCAT-R	TTCTCCGCATAGACCTTGAACTTTG	
SuSOD	SuSOD-F	TTTGTCCAAGAGGGAGATGG	Jain et al. [2015] [4]
	SuSOD-R	CTTCTCCAGCGGTGACATTT	

References

1. Singh, P.; Song, Q.Q.; Singh, R.K.; Li, H.B.; Solanki, M.K.; Malviya, M.K.; Verma, K.K.; Yang, L.T.; Li, Y.R. Proteomic analysis of the resistance mechanisms in sugarcane during *Sporisorium scitamineum* infection. *Int. J Mol. Sci.* **2019**, *20(3),* .569.
2. Reis, R.R.; da Cunha, B.A.D.B.; Martins, P.K.; Martins, M.T.B.; Alekcevetch, J.C.; Chalfun-Júnior, A.; Andrade, A.C.; Ribeiro, A.P.; Qin, F., Mizoi, J.; Yamaguchi-Shinozaki, K. Induced over-expression of AtDREB2A CA improves drought tolerance in sugarcane. *Plant Sci.* **2014,** *221*, 59-68.
3. Chen, S.S. Cloning and Expression Analysis of ROS Metabolism Pathway Key Genes from Sugarcane. Master's Thesis, Fujian Agriculture and Forestry University, Fuzhou, China, **2012**.
4. Jain, R.; Chandra, A.; Venugopalan, V.K.; Solomon, S. Physiological Changes and Expression of SOD and P5CS Genes in Response to Water Deficit in Sugarcane. *Sugar Tech*. **2015**, *17*, 276–282.

Supplementary Table S2. Morphological and biochemical characteristics of the isolate ASN-1

Tests	Isolate ASN-1
Gram reaction	+
Shape	Rod
Motility	+
Endospore	-
NaCl tolerance (%)	15
pH tolerance	10
Temperature tolerance	50
Catalase	+
Oxidase	+
Citrate utilization	+
Methyl red	+
Indole	+
Phenylalanine deamination	+
Nitrate Reduction	+
Lysine utilization	+
Voges Proskauer's	-
Ornithine utilization	-
H2S production	-

‘+’ positive for test; ‘- ‘negative for test

Supplementary Table S3: Number of substrates utilized by the strain ASN-1.

Chemical Guild	Total number of Substrates	Strain ASN-1
Sugars	27	15
Chemical sensitivity	23	13
Acidic pH	2	02
Sodium Chloride	3	03
Lactic acid	1	00
Hexose-PO_4	2	02
Amino acid	9	09
Hexose acid	9	07
Reducing Sugar	2	02
Carboxylic acids, esters and fatty acids	18	07

Supplementary Table S4: Substrate present in each well of Biolog Micro-Plate

Biolog well Serial No.	Carbon sources (GNIII)
A1	Negative Control
A2	Dextrin
A3	D-Maltose
A4	D-Trehalose
A5	D-Cellobiose
A6	Gentiobiose
A7	Sucrose
A8	D-Turanose
A9	Stachyose
A10	Positive Control
A11	pH 6
A12	pH 5
B1	D-Raffinose
B2	α-D-Lactose
B3	D-Melibiose
B4	β-Methyl-D-Glucoside
B5	D-Salicin
B6	N-Acetyl-D-Glucosamine
B7	N-Acetyl-β-DMannosamine
B8	N-Acetyl-D-Galactosamine
B9	N-AcetylNeuraminic Acid
B10	1% NaCl
B11	4% NaCl
B12	8% NaCl
C1	α-D-Glucose
C2	D-Mannose
C3	D-Fructose
C4	D-Galactose
C5	3-Methyl Glucose
C6	D-Fucose
C7	L-Fucose
C8	L-Rhamnose
C9	Inosine
C10	1% Sodium Lactate
C11	Fusidic Acid
C12	D-Serine
D1	D-Sorbitol
D2	D-Mannitol
D3	D-Arabitol
D4	myo-Inositol
D5	Glycerol
D6	D-Glucose-6-PO4
D7	D-Fructose-6-PO4
D8	D-Aspartic Acid
D9	D-Serine
D10	Troleandomycin
D11	Rifamycin SV
D12	Minocycline
E1	Gelatin
E2	Glycyl-L-Proline

E3	L-Alanine
E4	L-Arginine
E5	L-Aspartic Acid
E6	L-Glutamic Acid
E7	L-Histidine
E8	L-Pyroglutamic Acid
E9	L-Serine
E10	Lincomycin
E11	Guanidine HCl
E12	Niaproof 4
F1	Pectin
F2	D-Galacturonic Acid
F3	L-Galactonic Acid Lactone
F4	D-Gluconic Acid
F5	D-Glucuronic Acid
F6	Glucuronamide
F7	Mucic Acid
F8	Quinic Acid
F9	D-Saccharic Acid
F10	Vancomycin
F11	Tetrazolium Violet
F12	Tetrazolium Blue
G1	p-Hydroxy- Phenylacetic Acid
G2	Methyl Pyruvate
G3	D-Lactic Acid Methyl Ester
G4	L-Lactic Acid
G5	Citric Acid
G6	α-Keto-Glutaric Acid
G7	D-Malic Acid
G8	L-Malic Acid
G9	Bromo-Succinic Acid
G10	Nalidixic Acid
G11	Lithium Chloride
G12	Potassium Tellurite
H1	Tween 40
H2	γ-Amino-ButryricAcid
H3	α-Hydroxy- Butyric Acid
H4	β-Hydroxy-D,LButyricAcid
H5	α-Keto-Butyric Acid
H6	Acetoacetic Acid
H7	Propionic Acid
H8	Acetic Acid
H9	Formic Acid
H10	Aztreonam
H11	Sodium Butyrate
H12	Sodium Bromate
